# Harnessing citizen science through mobile phone technology to screen for immunohistochemical biomarkers in bladder cancer

**DOI:** 10.1038/s41416-018-0156-0

**Published:** 2018-07-11

**Authors:** Peter Smittenaar, Alexandra K. Walker, Shaun McGill, Christiana Kartsonaki, Rupesh J. Robinson-Vyas, Janette P. McQuillan, Sarah Christie, Leslie Harris, Jonathan Lawson, Elizabeth Henderson, Will Howat, Andrew Hanby, Gareth J. Thomas, Selina Bhattarai, Lisa Browning, Anne E. Kiltie

**Affiliations:** 1 Cancer Research UK, London, EC1V 4AD UK; 2 Cancer Research UK/MRC Oxford Institute for Radiation Oncology, University of Oxford, Oxford, OX3 7DQ UK; 3 Department of Population Health, University of Oxford, Oxford, OX3 7LF UK; 4 MRC Population Health Research Unit, University of Oxford, Oxford, OX3 7LF UK; 5 Cancer Research UK Cambridge Institute, University of Cambridge, Cambridge, CB2 ORE UK; 6 Leeds Institute of Cancer and Pathology (LICAP), St James’s University Hospital, Leeds, LS9 7TF UK; 7 Cancer Sciences Unit, University of Southampton Faculty of Medicine, Southampton, SO16 6YD UK; 8 Leeds Teaching Hospitals NHS Trust, St James’s Hospital, Leeds, LS7 9TF UK; 9 Department of Cellular Pathology, John Radcliffe Hospital, Oxford, OX3 9DU UK; 10 The NIHR Oxford Biomedical Research Centre, John Radcliffe Hospital, Oxford, OX3 9DU UK

**Keywords:** Predictive markers, Urological cancer

## Abstract

**Background:**

Immunohistochemistry (IHC) is often used in personalisation of cancer treatments. Analysis of large data sets to uncover predictive biomarkers by specialists can be enormously time-consuming. Here we investigated crowdsourcing as a means of reliably analysing immunostained cancer samples to discover biomarkers predictive of cancer survival.

**Methods:**

We crowdsourced the analysis of bladder cancer TMA core samples through the smartphone app ‘Reverse the Odds’. Scores from members of the public were pooled and compared to a gold standard set scored by appropriate specialists. We also used crowdsourced scores to assess associations with disease-specific survival.

**Results:**

Data were collected over 721 days, with 4,744,339 classifications performed. The average time per classification was approximately 15 s, with approximately 20,000 h total non-gaming time contributed. The correlation between crowdsourced and expert H-scores (staining intensity × proportion) varied from 0.65 to 0.92 across the markers tested, with six of 10 correlation coefficients at least 0.80. At least two markers (MRE11 and CK20) were significantly associated with survival in patients with bladder cancer, and a further three markers showed results warranting expert follow-up.

**Conclusions:**

Crowdsourcing through a smartphone app has the potential to accurately screen IHC data and greatly increase the speed of biomarker discovery.

## Introduction

Personalised medicine involves tailoring treatment to reflect a patient’s individual tumour characteristics. For this to be used routinely, we need to find biomarkers robustly associated with cancer prognosis or predictive of outcome following therapy.

Immunohistochemistry (IHC) is widely used for biomarker identification. To automate the staining and analysis process, IHC is often combined with tissue microarray (TMA) technology. TMAs position hundreds of small-diameter tissue samples in a physical array, which can be stained and scanned as one unit. This has made it possible to generate a large volume of IHC data from many patients relatively quickly. The analysis of IHC stained tissue is largely performed by the naked eye. Such ‘manual’ scoring of IHC is time consuming and requires several trained researchers or histopathologists to reach a consensus score. Automated analysis software is quickly gaining ground especially for the most common cancers and stain types. Though there is little doubt that such algorithms will eventually improve beyond human capability, currently automated IHC scoring algorithms are not applicable to all samples, and manual intervention is required for challenging or ambiguous cases.^1–3^ A major barrier to more accurate algorithms is the availability of large labelled data sets to train new generations of supervised algorithms. Crowdsourcing is one commonly used approach to generate such labels, essentially facilitating, rather than competing with, algorithms.

Recently, a number of projects have reported success in using crowdsourcing for the analysis of large data sets within a range of scientific disciplines, including biochemistry and biomedical research.^4–10^ ‘Cell Slider’ is one such study which aimed to address the rate-limiting step of IHC manual scoring (https://www.cellslider.net/). Here, untrained members of the public were able to accurately score IHC data, with participants achieving similar results to trained pathologists in cancer cell identification, oestrogen receptor (ER) status assignment and associations of ER status with clinical outcome in breast cancer. However, participants demonstrated a bias in terms of overestimating the number of cancer cells in an image, thus compromising the accuracy of IHC scoring.^8^ ‘Trailblazer’ was developed following Cell Slider to further test the ability of the public to score IHC data and to identify methodological improvements that could increase the accuracy of publicly generated scores. When given more comprehensive tutorials, the public was highly accurate in their detection of cancer and IHC scoring.^10^

Issues with crowdsourcing include the drop-off rate and inactivity in user participation. Recent crowdsourcing projects have attempted to increase the audience base to increase participants’ activity by integrating scientific tasks into games. Examples of such ventures include Foldit,^5^ Phylo,^6^ EteRNA^7^ and Fraxinus.^9^ Such crowdsourcing games have led to bona fide scientific discoveries and generated improvements in existing computational algorithms.^11^

We identified a number of proteins worthy of assessment for potential associations with clinical outcome in lung and bladder cancer and conducted IHC on TMAs containing tissue from patient tumour samples. Due to the use of radiotherapy as a treatment modality in MIBC, a number of proteins involved in the repair of DNA damage were assessed (RAD50, MRE11, p53, p21). CK20 and CK5/6 were included in this study as these immunostains have previously been used to distinguish basal and luminal MIBC subtypes.^12^ MRE11, p53, p21, TIP60 and Ki67 IHC have previously been investigated in MIBC and found to be potentially associated with survival and/or cancer progression.^13–20^ However, there is currently not enough evidence to conclude whether these proteins are valid biomarkers for DSS in MIBC. We decided to employ crowdsourcing scoring to analyse the large amount of data generated. A key objective was to retain the accuracy of manual scoring while being less time-consuming for the experts.

Scoring IHC data in its most basic form is a task involving pattern recognition and determination of colour gradients. We therefore hypothesised that, given a short tutorial, members of the public as a group would be able to accurately assess the IHC staining of cancerous tissue and that crowdsourcing could increase the speed of scoring large sets of IHC data. Unlike previous web-based crowdsourcing efforts in IHC scoring, we input our IHC data into a mobile gaming app available to members of the public. We first assessed the accuracy of the crowdsourced data. Then, when the crowdsourced scores were found to be accurate, we used these scores to look for associations between protein staining and clinical outcome.

## Materials and methods

### User recruitment

We crowdsourced the analysis of bladder cancer TMA core samples through the smartphone application (app) Reverse the Odds (RTO), distributed through Google Play and the iTunes store. Users in the app classified TMA samples as described below, and every so often were offered a separate minigame based on Reversi. TMA classifications were incentivised through powerups that could be used in the minigame. As such, during TMA classification there were no distractions, however the minigame provided some variety to an otherwise highly repetitive task. The majority of time was spent performing TMA classifications, though exact figures are unavailable. We did not store personal information about the users nor information regarding which user provided each classification. The data reported here were collected between 9th October 2014 and 28th September 2016.

### Tissue microarray samples

Ethical approval was obtained from London Bromley NRES (study 13/LO/0540), Leeds (East) Local Ethical Committee (studies 02/060 and 04/Q1206/62) and North West–Haydock Research Ethics Committee (study 14/NW/1033). Patients whose samples were collected from 2002 onwards gave informed consent for use of their pre-treatment biopsies.

All bladder cancer tissue cores were collected from four cohorts of patients. The first three were treated with radical radiotherapy at the Leeds Cancer Centre, UK (1995–2000, 2002–2005 and 2006–2009), and the remaining cohort was treated with radical cystectomy at the Leeds Teaching Hospitals NHS Trust, UK (1995–2005). The 1995–2005 cohorts have been previously described in Choudhury et al. (2010),^21^ and the 2006–2009 radiotherapy patients (*n* = 47) were treated as per the 2002–2005 radiotherapy cohort.

Haematoxylin and eosin (H&E)-stained sections from formalin-fixed paraffin embedded bladder tumour samples, taken at pre-treatment transurethral tumour resection, were reviewed by a consultant uropathologist (SB) and areas of invasive transitional cell carcinoma were outlined. Using a Beecher tissue microarrayer, 1356 0.6 µm cores were taken from up to five muscle-invasive areas per sample, and made into seven TMAs of up to 21 × 18 samples, including barrier samples of placenta, liver or mouse liver.

### Immunohistochemical staining

Lung cancer samples were stained for scoring in Reverse the Odds using anti-CD8 and AntiPDL1 antibodies and were entered into the game but not analysed further due to small number of responses and a shift in focus towards bladder cancer.

**Fig. 1:**
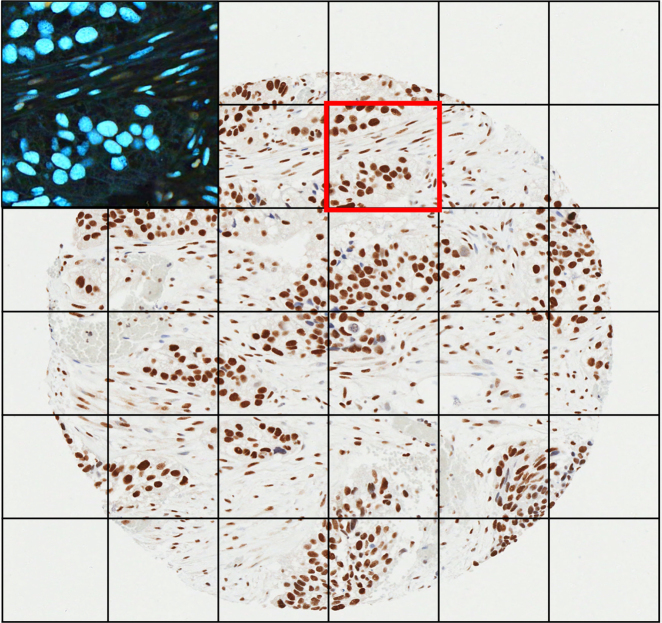
Typical 0.6 um TMA core, stained with DAB and haematoxylin counterstain, and split into 6 × 6 grid. Upper left panel shows contents of red bound square colour transformed for use in the app by citizen scientists

For bladder cancer, 11 different IHC stains were tested, using a BOND autostainer or manual methods. Details of the staining are given in Supplementary methods, with specific antibody conditions listed in Table S1. Slides were scanned using an Aperio ScanScope CS2 digital slide scanner at ×400 magnification and viewed using Aperio Image Scope viewing software. TMAs were then segmented using Aperio TMALab software. For use in the RTO app, the colours of the images were transformed from DAB and haematoxylin stained to inverted colours, to make scoring of the samples more appealing to the general public (Fig. 1). Cores were split into 36 segments to allow the user to comfortably inspect individual cells. The running order is shown in Table S2 and the results for the bladder cancer samples presented.

### Task design

Users were presented with a brief tutorial explaining how to spot cancerous tissue/cells, how to assess the proportion of cancer cells that were stained, and how to assess the intensity of staining (Fig. S1A-C). They were then presented with a segment and asked to score it (Fig. 1 and Fig. S1D, E). We only asked for the proportion of staining if cancer was indicated in the first question, and we only asked for intensity of staining if proportion was indicated as >0%. For the ‘proportion of cancer cells stained’ question we used 5 numerical ranges but used three different sets of ranges depending on the specific stain (Table 1): Category 1: 0, 1–25, 25–50, 50–75, 75–95, 95–100%; category 2: 0, 1–10, 10–25, 25–50, 50–75, 75–100%; category 3: 0, 1–10, 10–25, 25–65, 65–95, 95–100%. Users could access the instructions at any point during this process.

**Table 1:** Overview of markers, classifications and expert vs. crowdsourcing scores

Marker	Localisation	Total # cores	Total # classifications	Average # classifications per core	# Cores scored by experts (% of cores)	‘Proportion stained’ category	H-score, Spearman correlation with experts (95% CI)	Proportion cancer cells stained, Spearman correlation with experts (95% CI)	Intensity of staining, quadratic-weighted kappa with experts (95% CI)
MRE11 original	Nuclear	831	910,008	1095	88 (11%)	1	0.67 (0.52, 0.80)	0.44 (0.22, 0.63)	0.47 (0.30, 0.61)
RAD50	Nuclear	786	455,429	579	106 (13%)	1	0.81 (0.71, 0.88)	0.39 (0.19, 0.57)	0.73 (0.56, 0.85)
p21	Nuclear	845	1,040,933	1232	95 (11%)	1	0.90 (0.84, 0.93)	0.87 (0.80, 0.91)	0.57 (0.39, 0.72)
53BP1	Nuclear	841	201,390	239	85 (10%)	1	0.70 (0.55, 0.80)	0.53 (0.34, 0.68)	0.67 (0.52, 0.79)
p53	Nuclear	849	265,334	313	96 (11%)	2	0.92 (0.86, 0.95)	0.85 (0.77, 0.91)	0.69 (0.57, 0.79)
CK5/6	Membranous	814	86,469	106	104 (13%)	3	0.82 (0.71, 0.89)	0.66 (0.50, 0.78)	0.86 (0.77, 0.92)
CK20	Membranous	806	78,613	98	93 (12%)	3	0.88 (0.83, 0.92)	0.83 (0.75, 0.88)	0.87 (0.79, 0.92)
TIP60	Nuclear	850	90,850	107	91 (11%)	1	0.66 (0.52, 0.76)	0.17 (−0.07, 0.39)	0.59 (0.46, 0.70)
MRE11 new	Nuclear	524	49,857	95	84 (16%)	1	0.65 (0.49, 0.78)	0.61 (0.44, 0.75)	0.58 (0.41, 0.72)
MRE11 c-terminal	Nuclear	376	102,417	272	79 (21%)	1	0.79 (0.66, 0.86)	0.66 (0.49, 0.78)	0.70 (0.55, 0.80)
Ki67	Nuclear	775	68,637	89	86 (11%)	2	0.80 (0.72, 0.86)	0.80 (0.71, 0.87)	0.19 (−0.01, 0.37)

Classifications were performed on each segment of a core, with between 5 and 25 ratings per segment. These classifications were aggregated across segments to arrive at a single score per core. A subset of cores was scored by experts. The final three columns show correspondence between user and expert scores, presented as Spearman correlation (for H-score and proportion of cancer cells stained) or as quadratic-weighted kappa (for intensity of staining), with bootstrapped 95% CI. Proportion stained category 1: 0, 1–25, 25–50, 50–75, 75–95, 95–100%; category 2: 0, 1–10, 10–25, 25–50, 50–75, 75–100%; category 3: 0, 1–10, 10–25, 25–65, 65–95, 95–100%

### Gold standard scoring

Experts (AK and SMcG, with AW where consensus could not be achieved between two scorers) assessed the presence of cancer and proportion and intensity of staining in DAB-stained whole cores from TMAs to assess the performance of the crowdsourcing method. Gold standard scores were provided for whole TMA cores for approximately 10 to 15% of the data (Table 1).

### Statistical analysis

#### Aggregation of scores

We aggregated individual scores to arrive at a single proportion and intensity score for each core. Each core was scored between 81 and 3676 times across all of its segments (mean: 405, SD: 455, median: 124, IQR: [97, 1030]). Rather than aggregate responses first by segment and then by core, we took the mean across all proportion and intensity scores respectively for a core, ignoring any responses where the participant indicated there was no cancer. When a response indicated “no cancer”, this might be because they were shown a piece of the TMA without cancer cells. In that case we were correct to exclude their response from the aggregate. If the user did erroneously report “no cancer” for a sample with cancer, their response was incorrect and we were also correct to exclude it. As the dataset in some cases contained multiple cores per patient, we combined cores for each patient by taking the mean for proportion and intensity.

#### Linear correction of scores

Taking the (weighted) arithmetic mean across users and then segments, as done here, is an appropriate way of averaging out noisy scores, but only if errors are symmetrically distributed around the true mean. However, because the scores are bounded in this experiment, we would expect to consistently overestimate scores that are close to zero, and underestimate scores that are close to the maximum. For example, if the true proportion of cancer cells stained by the marker is 0, then ‘noisy’ individual users can only *overestimate* the score. As such, there are no *underestimates*—because a user cannot provide an answer that is smaller than 0—and the average user score is biased in a positive direction, i.e., it can only be ≥0.

To correct for this bias in proportion and intensity scores respectively, we applied a linear correction with clipping at the minimum and maximum values (e.g., any scores corrected by the linear model that ended up below 0 were set to 0). For the cores that had no expert scores, we could use all expert-scored cores to determine the intercept and slope between cores scored by both citizen scientists and experts. To correct the cores also scored by experts, we could not use the data that needed correction in the calculation of the very correction. This would lead to overly accurate crowdsourced scores. We therefore applied 10-fold cross-validation, using *sklearn.cross_validation.cross_val_predict*, described in Pedregosa et al. (2011)^22^ with a linear regression estimator. Any scores that were out-of-bounds of the original range were set to the bound (e.g., a corrected proportion of −5% was set to 0%). Critically, this approach ensures that the correction applied to a score was never based on the error of that score in the first place, such that any subsequent comparisons of expert and user scores were still valid.

After correcting the intensity and proportion metrics we calculated an H-score for each core as described in McCarty et al. (1985).^23^ We calculated this semi-quantitative score by multiplying the proportion of cancer cells stained by the marker (0 to 100%) by the average intensity of the staining (0 to 3). The H-score is therefore between 0 and 300, with 0 indicating no cancer cells positive for the marker, and 300 indicating all cancer cells positive with maximum intensity.

#### Comparison of user and expert scores

We calculated Spearman’s rank correlation between expert and crowdsourced scores for the H-score and proportion of cancer cells stained. For intensity of staining we used quadratic-weighted kappa.^24^

#### Associations with clinical outcomes

We used Kaplan–Meier curves and Cox proportional hazards models to estimate the associations between crowdsourced scores and disease-specific survival (DSS, time from treatment to death due to bladder cancer). For each marker, we examined the associations of the proportion of cancer cells staining positive, of the intensity of staining and of the H-score with DSS. Associations between H-score and survival were assessed using the numerical value of the H-score and quartiles for the H-scores. Quartiles were calculated on the combined dataset of all cohorts to ensure comparability of estimates for different cohorts. In the 2006–9 radiotherapy cohort, a significant number (>2/3rds) of cores were unusable due to diathermy artefacts within the tissue, created at the time of transurethral resection of the bladder tumour, therefore patient scores were unlikely to be representative, hence the 2006–9 cohort was not included in further analysis. The analysis was done separately in the cystectomy cohort and the two radiotherapy cohorts (1995–1999 and 2002–2005). The proportional hazards assumption was assessed by examining scaled Schoenfeld residuals. The main analyses were done using the aggregated scores. We carried out the analysis with and without adjustment for age, T stage, N stage, grade, sex and hydronephrosis. As a sensitivity analysis we repeated the main analyses with all observations of crowdsourced scores (from all individuals who used the app), taking into account that there are multiple observations per patient. We also investigated the associations of each marker with DSS in subgroups defined by low/high CK5 and CK20 expression, in the cystectomy cohort and in the combined radiotherapy cohort (1995–1999 and 2002–2005).

## Results

### Public engagement

The game went live on 9th October 2014 and data reported here are those deposited up to 28th September 2016. Data were collected over 721 days, with 148,349 app downloads. The total number of classifications was 4,744,339 (excluding lung and test cases), and the mean number of classifications per day was 6580. The average time per classification was approximately 15 s, an estimate based on the app analytics data. The total time contributed (# classifications × time per classification)—excluding the gaming element of the app—was approximately 20,000 h.

**Fig. 2:**
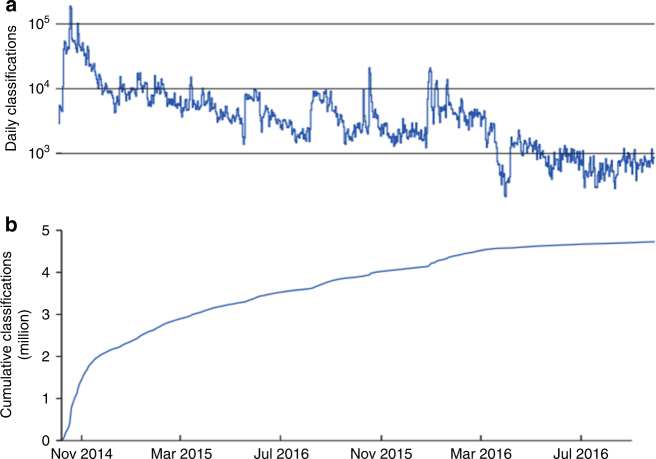
Plot of user participation over time. **a** Number of classifications per week. **b** Cumulative percentage of all classifications as a function of time

As typically observed for projects of this type, the rate of classifications altered markedly over the two years of play, increasing in response to marketing activity before returning to a low level “baseline” of activity. Marketing activity included paid-for social media and television advertising, as well as spontaneous news coverage and celebrity endorsements (Fig. 2). Activity level dropped in response to any technical issues preventing normal game function. In-game messaging or “push notifications” were employed to improve classification rate following the resolution of such issues. Generally, the classification rate lowered over time with over half of all classifications being scored in the first 4 months.

### Improving efficiency of crowdsourced scoring

**Fig. 3:**
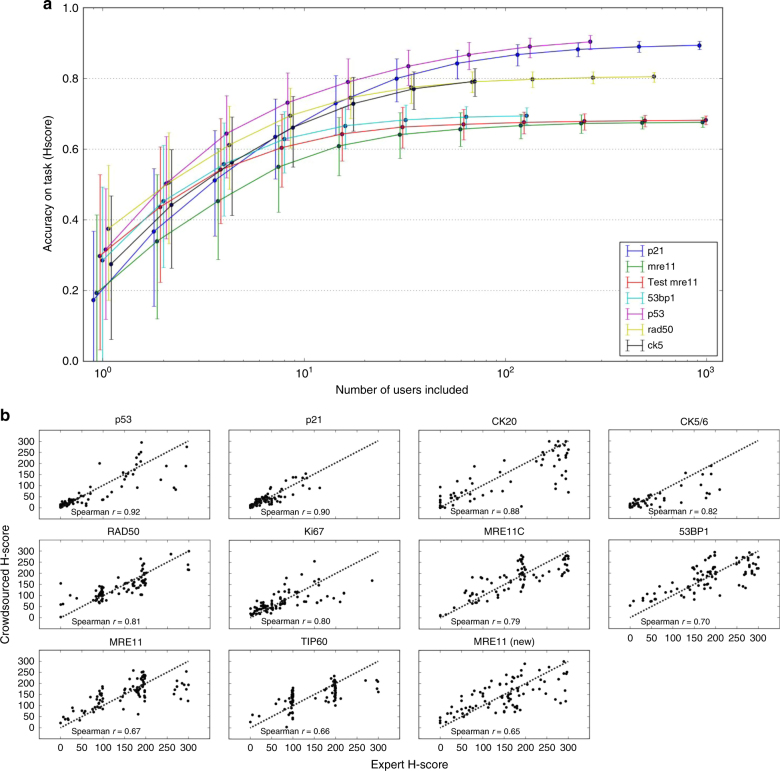
**a** Scatter plots for individual IHC stains ranked in order of H-score Spearman rho. *X*-axes represent the expert scores and *y*-axes the citizen score. Diagonal line represents a perfect score whereby the expert score is identical to the crowdsourced score; **b** The relationship between number of classifications and accuracy. The *y*-axis represents the H-score Spearman rho between expert and crowdsourced scores, and the x-axis represents the number of classifications used for a core. The accuracy is estimated through bootstrapping with 1000 samples. The error bars represent the bootstrapped 95% confidence interval (2.5 and 97.5 percentile of bootstrapped samples)

In early 2015, there was concern that users were finding it difficult to score the outer squares of a TMA core, which often contained only a handful of cells and therefore lacked the tissue structure that often helps distinguish cancer from non-cancer tissue. An analysis was performed using an MRE11 test set to assess the effect of scoring only the central 16 squares compared to all 36 squares. This revealed that these mostly empty segments could be discarded from analysis without detrimental effect on accuracy. Additionally, an interim analysis was performed in August 2015 to calculate accuracy of scoring as a function of the number of ratings per image. This was to see whether the datasets could be processed more quickly by reducing the number of raters from 25 per segment (Fig. 3a). Based on this figure it was decided that obtaining more than 5 ratings per segment, i.e., 80 ratings across the 16 segments of a core, would yield minimal additional accuracy. For example, with 80 raters per core the p21 stain is scored with an accuracy of 0.85. Having 1000 raters per core would yield an accuracy of 0.89. The trade-off, then, is to sacrifice 0.04 in accuracy to be able to analyse 12 times more stain types. Hence, we decided to use these additional ratings to analyse additional stains.

### Comparison of crowdsourced scores to expert scores

#### Proportion of cancer cells stained and intensity of staining

Although the H-score is the primary outcome of immunohistochemical analysis, examining citizen scientists’ accuracy on the proportion of cancer cells stained and the intensity of staining is instructive. We observed a wide range of correlations for both proportion and intensity, from 0.17 to 0.87 for proportion and 0.19 to 0.86 for intensity (Fig. S2 and Table 1). For example, estimating the proportion of cancer cells stained for TIP60 was difficult (Spearman correlation of 0.17 between crowdsourced and expert estimates), whereas the intensity of staining was poorly estimated for Ki67 (quadratic-weighted Kappa of 0.19 between crowdsourced and expert estimates). Other markers seemed considerably easier to score for the public, such as CK20 which was scored at an accuracy of >0.8 for both intensity of staining and proportion of cancer cells stained.

#### H-score accuracy

The H-score is a combination of the intensity and proportion estimates, and we calculated the correlation in H-score between expert and crowdsourced estimates (Fig. 3b and Fig. S3). There was no clear correlation with the time of the sample set entering into the game (Table S3). The correlation between crowdsourced and expert H-scores varied from 0.65 to 0.92 across the markers tested here, with six of 10 correlation coefficients at least 0.80.

### Associations between marker scores and disease-specific survival

Having established that crowdsourcing can yield reasonably accurate classifications of IHC scores, we then moved to see if any of these scores predicted disease-specific survival. Details of cohorts are shown in Tables S4 and S5. We fitted univariable Cox proportional hazards models for each of the stains. Statistically significant associations between H-score and DSS were found for MRE11, CK20, p21, 53BP1, p53 and Ki67 IHC (Table S6). Due to multiple testing some significant associations may be due to chance. However, MRE11 and CK20 displayed consistent relationships between IHC and DSS.

**Fig. 4:**
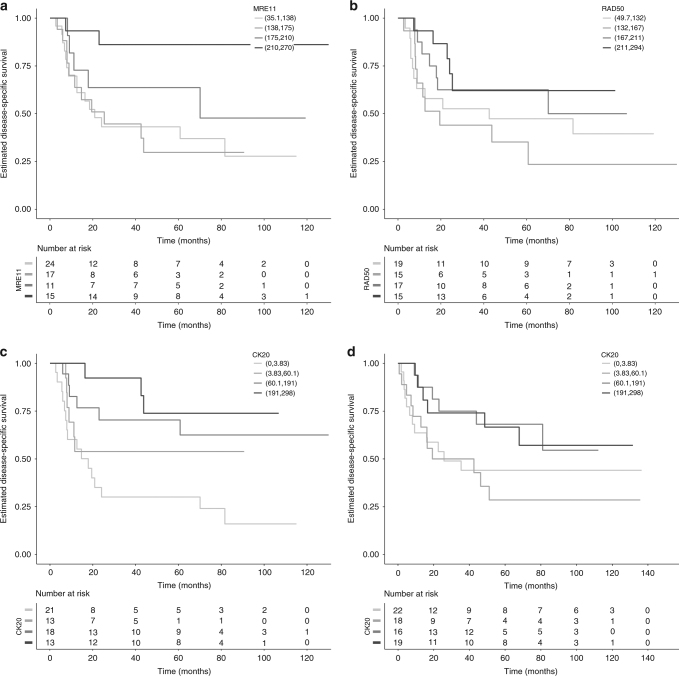
Kaplan Meier survival curves for disease-specific survival. (**a**) MRE11, (**b**) RAD50 and (**c**) CK20 for 1995-9 radiotherapy cohort, and (**d**) CK20 for cystectomy cohort

**Fig. 5:**
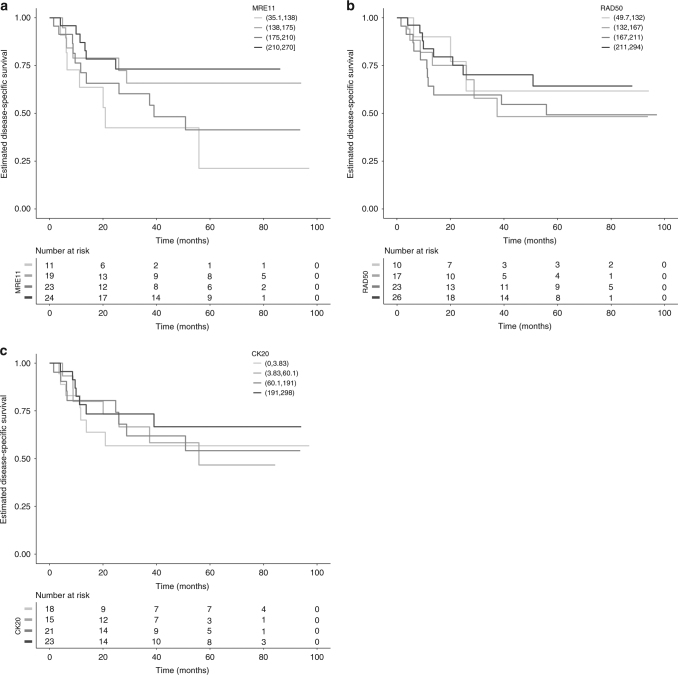
Kaplan Meier survival curves for disease-specific survival. (**a**) MRE11, (**b**) RAD50 and (**c**) CK20 for 2002-5 radiotherapy cohort

Similar to previously reported findings,^16, 21^ high MRE11 levels were found to be significantly associated with DSS in the radiotherapy cohorts but not the cystectomy cohort. Significance was observed in both the 1995–1999 and 2002–2005 radiotherapy cohorts when comparing the 1st quartile of H-scores to the 4th quartile (Table S6). Furthermore, when using a numeric H-score, rather than comparing quartiles, there was a significant association between MRE11 staining and DSS in the 1995–1999 cohort (HR per unit increase in H-score 0.991, 95% CI: 0.986–0.997, *p* = 0.004) and borderline-significant association in the 2002–2005 cohort (HR 0.994, 95% CI: 0.987–1.000, *p* = 0.060, Table S6, Figs. 4 and 5). High MRE11 expression (above its median) was not significantly associated with DSS in the cystectomy cohort. It was significantly associated with a lower risk of death due to bladder cancer in the radiotherapy 1995–1999 cohort (HR for high vs. low 0.30, 95% CI: 0.13–0.69, *p* = 0.004), but not in the 2002–2005 radiotherapy cohort, although in the same direction.

For CK20 staining, H-score was significantly associated with DSS in the cystectomy and 1995–1999 RT with a hazard ratio per unit increase for the 2002–2005 cohort of 0.998, 95% CI: 0.994–1.001, *p* = 0.20. CK20 levels above the median H-score were associated with improved survival in the cystectomy (HR: 0.454, 95% CI: 0.227–0.909, *p* = 0.026) and the 1995–9 (HR: 0.292, 95% CI: 0.134–0.638, *p* = 0.002, *n* = 65) cohorts (Figs. 4, 5 and S4).

Multivariable Cox proportional hazards models were fitted on the 1995–9 and 2002–5 radiotherapy cohorts for MRE11 and CK20 stains using age, T stage, N stage, grade, sex and hydronephrosis as covariables. The cystectomy cohort was excluded from multivariable analysis due to missing data. Results from multivariable analysis were in accordance with univariable analysis. In multivariable analysis for MRE11 staining in the 1995–9 cohort (*n* = 61) significant associations with DSS were identified for MRE11 H-score for unit increase (HR: 0.992, 95% CI: 0.984–1.000, *p* = 0.05) and hydronephrosis (HR: 3.101, 95% CI: 1.154–8.335, *p* = 0.02). In the 2002–2005 cohort (*n* = 73) only MRE11 H-score was significantly associated with DSS (HR: 0.990, 95% CI: 0.981–0.998, *p* = 0.02). In multivariable analysis of CK20 staining in the 1995–1999 cohort (*n* = 59), a significant association was observed for CK20 H-score and DSS (HR: 0.993, 95% CI: 0.990–1.000, *p* = 0.02). In the 2002–2005 cohort (n = 74) no significant association was found for CK20 or any of the variables analysed.

In the combined radiotherapy cohort, in the group with low CK5 expression (less than its median) CK20 and MRE11 were associated with a lower risk of bladder cancer death (HR per unit increase 0.994, 95% CI: 0.990–0.998, *p* = 0.003, and 0.991 95% CI: 0.985–0.996, *p* = 0.0007, respectively). In the high CK5 subgroup the associations were similar in direction and magnitude but only borderline significant. In the high CK20 subgroup, 53BP1 was associated with a lower risk of bladder cancer death (HR: 0.992, 95% CI: 0 .985 = 0.999), *p* = 0.02). MRE11 was also associated with a lower risk (HR: 0.989, 95% CI: 0.981–0.997, *p* = 0.007). None of the markers were significantly associated with outcome in the low CK20 group.

## Discussion

Reverse the Odds was a novel approach aimed at improving the speed of IHC scoring. Mobile gaming technology was combined with crowdsourcing to bring citizen science to a wider user-base than other projects such as Cancer Research UK’s *Cell Slider* and *Trailblazer*. We observed moderate to high agreement between crowdsourced and expert scores, and crowdsourced scores successfully identified at least two markers as predictive of survival in bladder cancer.

As implemented in this study, our approach did not increase the speed of IHC scoring, as initially anticipated. It took just under 2 years to score 16 IHC markers, far longer than it would have taken researchers. However, the use of crowdsourcing embedded in mobile phone technology is in its infancy, and throughout the course of RTO lessons were learnt that would speed up analysis more than 10-fold for future projects.

In early 2015, there was concern that users were finding it difficult to score the outer squares of a TMA core, which often contained only a handful of cells and therefore lacked the tissue structure that often helps distinguish cancer from non-cancer tissue. Reducing the number of segments to be scored for each core to the 16 central segments did not affect the accuracy of core scoring and increased the speed at which a core could be scored by the public. Furthermore, accurate results could be achieved with fewer users than initially thought. With these adjustments, it is estimated that RTO could have analysed all 11 bladder cancer stains in the first 2 weeks after release of the game, which would amount to a significant increase in scoring efficiency over traditional scoring methods.

One problem identified in RTO was the drop-off in user participation over time. Indeed, half of all image analysis was conducted in the first 4 months of the game’s release (Fig. 2). This pattern has been observed in other crowdsourcing ventures such as *Galaxy Zoo, Milky Way Project, Fraxinus, EteRNA, Foldit* and *Phylo*. It is often found that a small group of dedicated individuals contribute the bulk of classifications, with the majority of users only contributing transiently, and with some registered users never actually participating.^9, 11, 25^ To exploit the high uptake and number of analyses conducted in crowdsourcing applications after initial release, good systems need to be in place upfront, and it is important that a robust user engagement plan for the promotion of a project is in place from the outset.

A major issue in using crowdsourcing in molecular pathology studies is its reliability. In RTO we found the accuracy of public scoring to vary between immunostains. The lowest accuracy in public scores was seen in stains which were classed by experts as more difficult. Some stains were particularly challenging, e.g., MRE11 and TIP60 which were scored with the lowest accuracy when compared to experts. MRE11 and TIP60 both show heterogeneous staining and can have weak non-specific staining in negative cells. Additionally, TIP60 IHC can also produce high levels of non-specific background staining in some cores. This reflects the potential need for improving contributor skills and ongoing quality assurance in such crowdsourcing projects. Previous work suggests that though tutorials can have short-term beneficial effects, a more critical predictor of accuracy is long-term engagement and training through experimentation and ongoing feedback.^26, 27^

Similar to *Cell Slider*,^8^ in RTO the public were asked to score an isolated segment of a TMA core. This step was taken to allow easy viewing of individual cells on a smartphone and eliminated the poor user experience of having to pinch-zoom. However, this user interface had the potential to limit the accuracy of scores generated by participants. The density of cancer cells can vary markedly across a TMA core. Using segmentation, the score from an area containing relatively few cancer cells is equal to the score derived from an area with a large number of cancer cells and has the potential to skew results, especially in cores where staining is heterogeneous. In this study we accepted this as additional noise, but future studies could account for such effects of segmentation through segment weighting. Furthermore, in viewing a whole tissue core, a scorer can get a ‘global’ view of the staining across the whole core and what level of staining most of the cancer cells exhibit, which can aid in the accuracy of scoring. Another benefit of viewing a complete tissue core is that it can help in distinguishing cancer from normal tissue and infiltrative lymphocytes. Despite these drawbacks of using a small mobile screen, we considered the trend from desktop- and laptop-based internet use towards mobile use sufficiently strong to explore the viability of a smartphone-based solution.

In our analysis of the association of public scores with clinical outcome we observed that there were multiple unusable cores in our 2006–9 radiotherapy cohort. This was due to the TMA block having been used for previous studies, unlike the others. The result of previous sectioning from the TMA was a loss of total cores from the TMA, an increase in cores lacking tumour tissue and an increase in cores affected by diathermy artefacts arising from the transurethral bladder tumour resection. Corresponding to this, when MRE11 staining was re-optimised and new sections stained, there were only 524 cores suitable for analysis compared to the 831 originally stained cores cut from less depleted blocks. We therefore question the utility of TMAs for muscle-invasive bladder cancer, as it is likely that only the top sections of a TMA will give reliable, representative results.

In terms of the association of public scores with clinical outcome, MRE11 and CK20 staining showed the strongest associations with DSS. This is an encouraging result as these two proteins have been linked to DSS in MIBC previously.

MRE11 IHC has been directly reported to associate to DSS in MIBC by two independent research groups.^16, 21^ Both studies identified high levels of MRE11 (greater than the 1st quartile of the data) to be associated with improved DSS in MIBC following radiotherapy-based treatment but with no association with outcome following cystectomy. While the results of this present analysis only found MRE11 levels above the 4th quartile to be significantly associated with improved DSS, the public scores were accurate enough to at least identify MRE11 as a candidate marker for further investigation.

CK20 has been used to identify the luminal subtype of MIBC. Luminal MIBC is associated with better DSS compared to basal MIBC^12^ and hence CK20 may be a potential prognostic biomarker for MIBC. In this study, the crowdsourced CK20 scores identified a significant association between higher levels of CK20 and improved patient outcome in both the cystectomy and the 1995–1999 radiotherapy cohort in keeping with CK20 being a prognostic marker for MIBC. In contrast, crowdsourced scores for the basal marker CK5/6 were not associated with DSS in this analysis, despite good agreement between experts and crowdsourced scores. This is surprising given that high levels of CK5/6 being previously associated with poor DSS.^28–30^

In this study, we have gained insight into the potential advantages and disadvantages of using crowdsourcing for the analysis of molecular pathology studies. A major advantage of using crowdsourcing to analyse IHC data is the potential time a well-planned and optimised method could save for skilled researchers. For crowdsourcing, researchers would only be required to score a small subset of data to generate tutorial images and 10% comparison data, thus freeing up time for other work. Although RTO did not achieve improvements in scoring efficiency, a number of steps could have been taken to dramatically speed up analysis. First, all datasets can be prepared in advance (preprocessing, segmentation, colour inversion, and hosting) to rapidly switch to a new dataset once a previous set is completed. Such completions happen in sometimes unpredictable bursts e.g., due to media coverage. Second, prior to any media launch every effort should be made to optimise the number of ratings that are necessary per sample. Many ratings happen upon initial launch, and these are in effect wasted if too many ratings are collected per sample. In RTO, prior optimisation of number of raters as outlined in the results would have seen all datasets analysed within the first 14 days of launch. Third, a community should be fostered to encourage learning and continued engagement, dramatically increasing retention of users obtained through marketing. Fourth, microtasks could be distributed to users based on their ability as assessed through scoring expert-scored samples. If the lessons learnt from RTO and *Trailblazer* are applied to future projects, crowdsourcing has the potential to accurately screen IHC data and greatly increase the speed of biomarker discovery from large IHC data sets.

## Electronic supplementary material

Supplementary material

## Availability of data and material

The datasets generated and/or analysed during the current study are available from the corresponding author on reasonable request.
