# Supplementary material for: Harnessing citizen science through mobile phone technology to screen for immunohistochemical biomarkers in bladder cancer
Source: Br J Cancer. 2018 Jul 11;119(2):220–9. doi: 10.1038/s41416-018-0156-0 (PMC6048059; doi:10.1038/s41416-018-0156-0)
Supplement: Supplementary file 1 — Supplementary material [file 41416_2018_156_MOESM1_ESM.docx]

**Supplementary data**

**Supplementary methods**

Immunohistochemical staining

Lung cancer samples were stained for scoring in Reverse the Odds using anti-CD8 and AntiPDL1 antibodies and were entered into the game but not analysed further due to small number of responses and a shift in focus towards bladder cancer.

For bladder cancer, 11 different IHC stains were tested, using a BOND autostainer or manual methods. The manual methods for p21 and p53 staining, using a standard avidin-biotin-peroxidase technique, are as previously described (Cazier *et al* 2014). Manual staining of MRE11c (an antibody to the C-terminal of MRE11 (ab30725, abcam)) was performed using the Novolink polymer detection system using manufacturer's guidelines with heat-induced epitope retrieval conducted in a low pH buffer at 110^o^C for 45 seconds using a decloaking chamber. Primary antibody was diluted 1:1000 in kit specific antibody diluent and incubated on samples for 1 hour at room temperature. Automated staining was conducted using the Leica Bond-max™ stainer with Leica Bond Polymer Refine Detection Kit (DS9800) and assay specific reagents. Standardised conditions were used for deparaffinisation, rehydration, peroxidase blocking, post primary and polymer incubation steps. Primary antibody was diluted in 1% Bovine serum albumin (BSA, VWR International, Leicestershire, UK) for all staining protocols except “MRE11 (new)” which utilised 10% BSA. Primary antibody incubation was conducted at room temperature. Specific antibody conditions are shown in Table S1. Two datasets for MRE11 IHC staining were input into RTO. This was due to a re-optimisation of the MRE11 staining protocol which occurred after the first dataset was input into the app. The re-optimised MRE11 staining was called MRE11 (new).

Slides were scanned using an Aperio ScanScope CS2 digital slide scanner at x400 magnification and viewed using Aperio Image Scope viewing software. TMAs were then segmented using Aperio TMALab software. Digital images of cores could then be downloaded with their own unique core ID. For each stain, cores were selected for study if there was no diathermy damage and tumour tissue was present in sufficient quantity for scoring. Training and advice were given by a Consultant Uropathologist (LB).

For use in the RTO app, the colours of the images were transformed from DAB and haematoxylin stained to inverted colours, to make scoring of the samples more appealing to the general public (Figure 1). Furthermore, the cores were not presented whole, but rather split into 36 segments along a 6x6 square grid. In later versions of the app we further excluded the 20 segments along the outside of the image, which were often empty or had few cells present. The use of segments rather than whole cores allowed the user to comfortably inspect individual cells on a smartphone without zooming. However, this approach lacked a whole core view which could have helped distinguish cancer from non-cancer tissue. The running order for the various stains is shown in Table S2. The results for the bladder cancer samples are presented.

Cazier J-B, Rao SR, McLean CM, Walker AK, Wright BJ, Jaeger EE, *et al*. Whole-genome sequencing of bladder cancers reveals somatic CDKN1A mutations and clinicopathological associations with mutation burden. *Nature Communications* 2014; **5**: 4809.

**Table S1: Immunohistochemistry staining parameters for Bond-max Stainer**

| **Antibody** | **Dilution** | **Heat Induced Epitope retrieval at 100^o^C** | **Conditions** |
| --- | --- | --- | --- |
| Mre11 ab214 (Abcam)  (original) | 1:6000 | Epitope retrieval solution 1 - 20 minutes | Primary antibody incubation - 15minutes |
| Mre11 ab214 (Abcam) (new) | 1:6000 | Epitope retrieval solution 1 - 20 minutes | Pre-primary protein block -10% BSA for 30 minutes  Primary antibody incubation - 8 minutes |
| Tip60 nb100-87055 (Novus Biologicals) | 1: 400,000 | Epitope retrieval solution 1 - 20 minutes | Primary antibody incubation - 15minutes |
| Rad50 ab89 (Abcam) | 1:500 | Epitope retrieval solution 1 - 20 minutes | Primary antibody incubation - 15minutes |
| 53BP1 #4937 (Cell Signalling Technology) | 1:50 | Epitope retrieval solution 1 - 40 minutes | Primary antibody incubation – 30 minutes |
| Ki67 M7240 (Dako) | 1:1000 | Epitope retrieval solution 1 - 20 minutes | Primary antibody incubation – 30 minutes |
| CK5/6 M7237 (Dako) | 1:500 | Epitope retrieval solution 2 - 20 minutes | Primary antibody incubation – 30 minutes |
| CK20 E16444 (Immunologic) | 1:1000 | Epitope retrieval solution 1 - 20 minutes | Primary antibody incubation – 30 minutes |

**Table S2: Running order for data sets within Reverse the Odds**

| **Running order** | **Tumour type** | **Marker** |
| --- | --- | --- |
| 1 | Lung squamous cell* | CD8 (lymphocytes) |
| 2 | Lung adenocarcinoma* | CD8 (lymphocytes) |
| 3 | Bladder | MRE11 |
| 4 | Bladder | RAD50 |
| 5 | Lung adenocarcinoma* | Adeno Lung PDL1 |
| 6 | Bladder* | TEST MRE11 |
| 7 | Lung squamous cell* | Squamous Lung PDL1 |
| 8 | Bladder | p21 |
| 9 | Bladder | 53BP1 |
| 10 | Bladder | p53 |
| 11 | Bladder | CK5/6 |
| 12 | Bladder | CK20 |
| 13 | Bladder | TIP60 |
| 14 | Bladder | MRE11new (re-stained) |
| 15 | Bladder | MRE11c |
| 16 | Bladder | Ki67 |

*results not reported due to small sample size

**Table S3: Time of sample set entering the game and effect on Spearman correlation between expert and crowdsourced scores.**

| Order of addition to app |  |  | H-score correlation |
| --- | --- | --- | --- |
| 1 | Lung squamous cell | CD8 (lymphocytes) |  |
| 2 | Lung adenocarcinoma | CD8 (lymphocytes) |  |
| 3 | Bladder | MRE11 | 0.67 |
| 4 | Bladder | RAD50 | 0.81 |
| 5 | Lung adenocarcinoma | Adeno Lung PDL1 |  |
| 6 | Bladder | TEST MRE11 |  |
| 7 | Lung squamous cell | Squamous Lung PDL1 |  |
| 8 | Bladder | p21 | 0.9 |
| 9 | Bladder | 53BP1 | 0.7 |
| 10 | Bladder | p53 | 0.92 |
| 11 | Bladder | CK5/6 | 0.82 |
| 12 | Bladder | CK20 | 0.88 |
| 13 | Bladder | TIP60 | 0.66 |
| 14 | Bladder | MRE11new (re-stained) | 0.65 |
| 15 | Bladder | MRE11c | 0.79 |
| 16 | Bladder | Ki67 | 0.8 |

**Table S4:** Follow-up time (in months) by cohort.

|  | Minimum | 1^st^ quartile | Median | Mean | 3^rd^ quartile | Maximum |
| --- | --- | --- | --- | --- | --- | --- |
| RT 1995-2002 | 2.7 | 11.2 | 28.2 | 42.7 | 73.8 | 130.1 |
| RT 2002-2005 | 1.58 | 11.40 | 28.45 | 38.69 | 65.31 | 97.08 |
| RT2006-2009 | 1.68 | 13.65 | 30.09 | 29.03 | 43.88 | 65.05 |
| Cystectomy | 0.76 | 10.59 | 41.63 | 49.77 | 83.50 | 136.40 |

**Table S5**: Number of events by cohort. ‘Died of bladder cancer’ was the event of interest.

|  | Alive, bladder cancer-free | Alive with bladder cancer | Died of bladder cancer | Died of other causes |
| --- | --- | --- | --- | --- |
| RT 1995-2002 | 12 | 3 | 35 | 19 |
| RT 2002-2005 | 19 | 1 | 31 | 30 |
| RT2006-2009 | 17 | 3 | 17 | 7 |
| Cystectomy | 25 | 1 | 36 | 14 |

**Table S6. Clinical data and univariable analysis**

|  | **Cystectomy cohort** | **1995-1999 cohort** | **2002-2005 cohort** |
| --- | --- | --- | --- |
|  | Hazard ratio, [95% confidence interval, p value] | Hazard ratio, [95% confidence interval, p value] | Hazard ratio, [95% confidence interval, p value] |
| **Mean age, years (sd)** | 66.7 (7.7) | 73.1 (10.4) | 76.8 (7.2) |
| **Gender**  Male (%)  Female (%) | 57 (75)  19 (25) | 52 (75)  17 (24) | 61 (75)  20 (25) |
| **Hydronephrosis (%)** | 0 (0) | 10 (14) | 28 (35) |
| **MRE11** | n=75 | n=67 | n=77 |
| H-score (per unit increase) | 0.997 [0.991- 1.004, 0.41] | 0.991 [0.986- 0.997, **0.004****] | 0.993 [0.987-1.00, 0.06] |
| 1^st^ vs 2^nd^ quartile | 1.808 [0.721- 4.534, 0.21] | 1.041 [0.477- 2.273, 0.92] | 0.374 [0.125- 1.119, 0.08] |
| 1^st^ vs 3^rd^ quartile | 1.4124 [0.547- 3.649, 0.48] | 0.550 [0.200- 1.516, 0.25] | 0.6814 [0.263- 1.764, 0.43] |
| 1^st^ vs 4^th^ quartile | 0.5646 [0.1788- 1.783, 0.33] | 0.141 [0.032- 0.620, **0.009****] | 0.283 [0.095- 0.847, **0.024***] |
| **RAD50** | n=71 | n=66 | n=76 |
| H-score (per unit increase) | 1.003 [0.995-1.012, 0.44] | 0.995 [0.989-1.002, 0.15] | 0.998 [0.991-1.006, 0.66] |
| 1^st^ vs 2^nd^ quartile | 1.2285 [0.577- 2.616, 0.60] | 1.355 [0.569- 3.224, 0.49] | 1.508 [0.390- 5.837, 0.55] |
| 1^st^ vs 3^rd^ quartile | 0.6223 [0.205- 1.892, 0.40] | 0.656 [0.253- 1.700, 0.38] | 1.650 [0.459- 5.931, 0.44] |
| 1^st^ vs 4^th^ quartile | 3.7724 [0.809- 17.581, 0.09] | 0.484 [0.167- 1.404, 0.18] | 0.998 [0.264- 3.767, 0.998] |
| **p21** | n=75 | n=67 | n=76 |
| H-score (per unit increase) | 1.003 [0.994-1.012, 0.47] | 0.990 [0.976-1.003, 0.13] | 1.006 [0.997-1.015, 0.19] |
| 1^st^ vs 2^nd^ quartile | 2.569 [1.105- 5.974, **0.028***] | 2.129 [0.936- 4.844, 0.07] | 1.056 [0.366- 3.046, 0.92] |
| 1^st^ vs 3^rd^ quartile | 1.313 [0.500- 3.449, 0.58] | 0.555 [0.186- 1.659, 0.29] | 0.985 [0.331- 2.935, 0.98] |
| 1^st^ vs 4^th^ quartile | 1.8584[0.706- 4.889, 0.201] | 0.633 [0.170- 2.355, 0.50] | 1.835 [0.331- 2.935, 0.25] |
| **53BP1** | n=74 | n= 67 | n=77 |
| H-score (per unit increase) | 0.997 [0.988-1.007, 0.57] | 0.995 [0.988-1.001, 0.10] | 0.995 [0.988-1.002, 0.15] |
| 1^st^ vs 2^nd^ quartile | 0.697 [0.326- 1.489, 0.35] | 1.347 [0.505- 3.595, 0.55] | 0.613 [0.222- 1.694, 0.34] |
| 1^st^ vs 3^rd^ quartile | 0.703 [0.277- 1.787, 0.46] | 0.840 [0.330- 2.140, 0.71] | 0.951 [0.366- 2.471, 0.92] |
| 1^st^ vs 4^th^ quartile | 5.061 [1.080- 23.717, **0.04***] | 0.480 [0.174- 1.328, 0.16] | 0.4203 [0.137- 1.291, 0.13] |
| **p53** | n=73 | n= 67 | n=77 |
| H-score (per unit increase) | 0.999 [0.996-1.003, 0.66] | 1.000 [0.995-1.004, 0.91] | 1.002 [0.997-1.007, 0.51] |
| 1^st^ vs 2^nd^ quartile | 6.165 [2.014- 18.871, **0.001*****] | 0.623 [0.226- 1.718, 0.36] | 6.709 [1.899- 23.707, **0.003****] |
| 1^st^ vs 3^rd^ quartile | 1.428 [0.403- 5.063, 0.58] | 1.221 [0.518- 2.878, 0.65] | 2.6069 [0.690- 9.848, 0.16] |
| 1^st^ vs 4^th^ quartile | 2.635 [0.837- 8.301, 0.10 ] | 0.674 [0.244- 1.858, 0.44] | 4.0597 [1.007- 16.366, 0.05] |
| **CK5/6** | n=75 | n=67 | n=77 |
| H score (per unit increase) | 1.002 [0.997-1.007, 0.41] | 1.001 [0.995-1.008, 0.75] | 0.999 [0.992-1.006, 0.76] |
| 1^st^ vs 2^nd^ quartile | 1.906 [0.411- 8.827, 0.41] | 1.066 [0.484- 2.351, 0.87] | 0.762 [0.263- 2.206, 0.62] |
| 1^st^ vs 3^rd^ quartile | 1.187 [0.265- 5.309, 0.82] | 0.589 [0.169- 2.054, 0.41] | 0.556 [0.169- 1.824, 0.33] |
| 1^st^ vs 4^th^ quartile | 0.349 [0.320- 6.289, 0.65] | 0.780 [0.280- 2.168, 0.63] | 0.8194 [0.297- 2.260, 0.70] |
| **CK20** | n=75 | n=65 | n=77 |
| H-score (per unit increase) | 0.996 [0.993-1.000, **0.05 ***] | 0.992 [0.988-0.997, **0.002****] | 0.998 [0.994-1.001, 0.20] |
| 1^st^ vs 2^nd^ quartile | 1.209 [0.542- 2.695, 0.64] | 0.521 [0.203- 1.339, 0.18] | 0.982 [0.344- 2.805, 0.97] |
| 1^st^ vs 3^rd^ quartile | 0.4941 [0.185- 1.319, 0.16] | 0.291 [0.114- 0.746, **0.01***] | 0.921 [0.333- 2.543, 0.87] |
| 1^st^ vs 4^th^ quartile | 0.4997 [0.187- 1.333, 0.17] | 0.169 [0.049- 0.581, **0.005***] | 0.686 [0.241- 1.958, 0.48] |
| **Tip60** | n=76 | n=68 | n=75 |
| H-score (per unit increase) | 0.997 [0.988-1.006, 0.51] | 0.999 [0.990-1.009, 0.89] | 1.000 [0.989-1.010, 0.95] |
| 1^st^ vs 2^nd^ quartile | 0.512 [0.212- 1.233, 0.14] | 1.609 [0.652- 3.967, 0.30] | 0.852 [0.286- 2.537, 0.77] |
| 1^st^ vs 3^rd^ quartile | 0.756 [0.298- 1.918, 0.56] | 0.803 [0.305- 2.111, 0.66] | 0.593 [0.208- 1.694, 0.33] |
| 1^st^ vs 4^th^ quartile | 0.679 [0.268- 1.723, 0.42] | 1.538 [0.604- 3.915, 0.37] | 0.705 [0.255- 1.951, 0.50] |
| **MRE11 new** | n=58 | n=62 | n=74 |
| H-score (per unit increase) | 1.002 [0.995-1.009, 0.645] | 0.995 [0.989-1.001, 0.11] | 1.000 [0.994-1.007, 0.91] |
| 1^st^ vs 2^nd^ quartile | 1.354 [0.524- 3.501, 0.53] | 0.965 [0.379- 2.455, 0.94] | 2.114 [0.236- 18.944, 0.50] |
| 1^st^ vs 3^rd^ quartile | 1.092 [0.304- 3.923, 0.89] | 0.643 [0.225- 1.840, 0.41] | 1.034 [0.107- 9.959, 0.98] |
| 1^st^ vs 4^th^ quartile | 1.731 [0.382- 7.839, 0.48] | 0.411 [0.120- 1.407, 0.16] | 1.293 [0.162- 10.352, 0.81] |
| **MRE11c** | n=23 | n=62 | n=33 |
| H-score (per unit increase) | 0.997 [0.986-1.009, 0.67] | 0.999 [0.993-1.005, 0.76] | 1.012 [0.995-1.029, 0.18] |
| 1^st^ vs 2^nd^ quartile | NA* | 0.714 [0.268- 1.904, 0.50] | NA |
| 1^st^ vs 3^rd^ quartile | NA | 0.498 [0.180- 1.377, 0.18] | NA |
| 1^st^ vs 4^th^ quartile | NA | 1.258 [0.450- 3.520, 0.66] | NA |
| **Ki67** | n= 74 | n=65 | n=75 |
| H-score (per unit increase) | 0.994 [0.985-1.003, 0.21] | 1.004 [0.997-1.012, 0.25] | 0.991 [0.980-1.002, 0.11] |
| 1^st^ vs 2^nd^ quartile | 0.293 [0.105- 0.819, **0.019***] | 0.503 [0.175- 1.441, 0.20] | 0.464 [0.189- 1.139, 0.09] |
| 1^st^ vs 3^rd^ quartile | 0.557 [0.247- 1.257, 0.16] | 0.489 [0.154- 1.556, 0.23] | 0.258 [0.074- 0.896, 0.03] |
| 1^st^ vs 4^th^ quartile | 0.572 [0.229- 1.430, 0.23] | 1.277 [0.508- 3.209, 0.60] | 0.292 [0.084- 1.008, 0.05] |


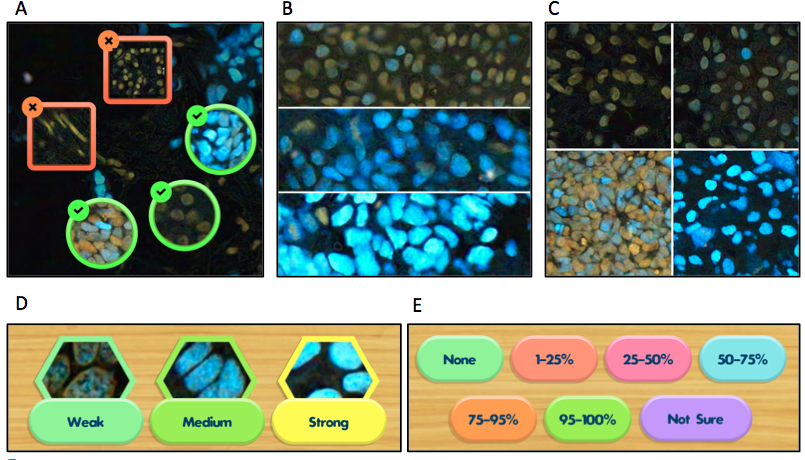


**Figure S1: Example tutorial embedded in the game and buttons used to score segments during the game.** **(A)** Tutorial panel demonstrating tumour cells stained for p53 to score (surrounded by green circles and tick) and stromal cells (lower red box with cross) and lymphocytes (upper red box with cross) not to be scored. **(B)** Panels demonstrating range of intensities from 1+ to 3+ (top to bottom). **(C)** Panels demonstrating a range of proportions of cells stained. **(D)** When scoring, players were asked to click on the button most closely representing the section presented for intensity: weak (1+), medium (2+) and strong staining (3+). **(E)** When scoring, players were asked to click on the button most closely representing the section presented for proportion of cancer cells stained. Percentage bins varied depending on the data set being used (See Table 2).


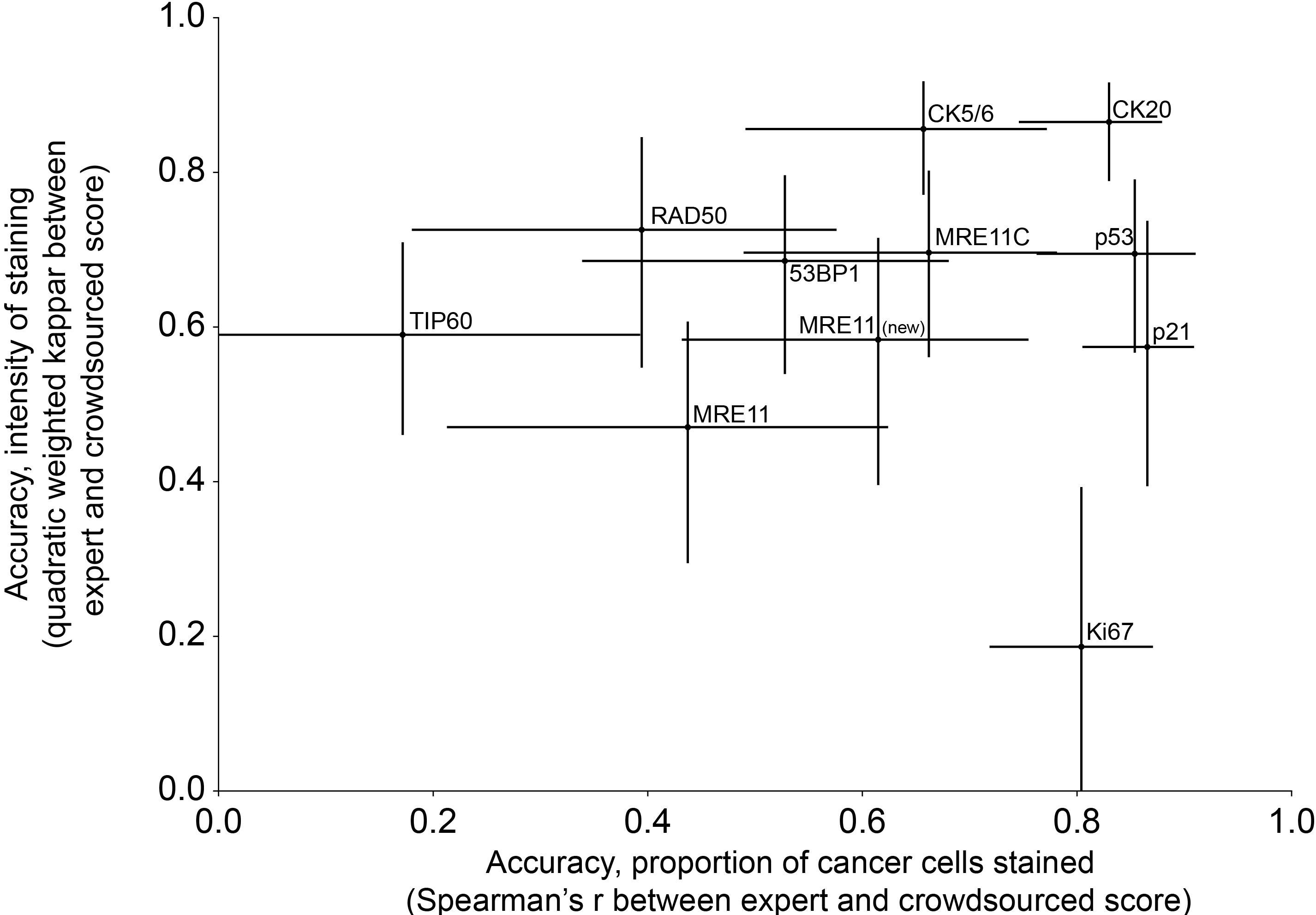


**Figure S2: Accuracy of marker proportion and intensity across each of 11 markers.** The x-axis shows the accuracy of citizen scientists in estimating the proportion of cancer cells stained by the marker, expressed as Spearman correlation with expert scores. The y-axis shows the quadratic-weighted kappa between citizen scientist and expert scores for the intensity of staining. Error bars represent bootstrapped 95% CI.


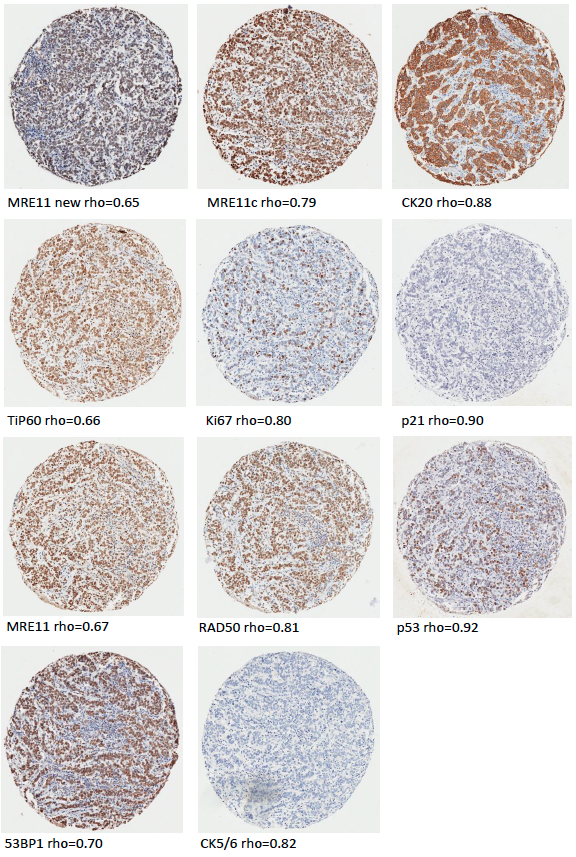


**Figure S3: Example staining pattern of antibodies, and corresponding Spearman’s correlation coefficients for each sample type.** Right-hand column correlation 0.88 to 0.92; middle column 0.79-0.82; left-hand column 0.65 to 0.70.

**A)**


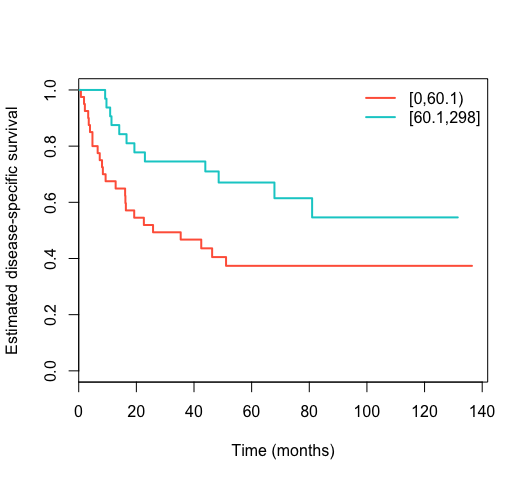


**B)**


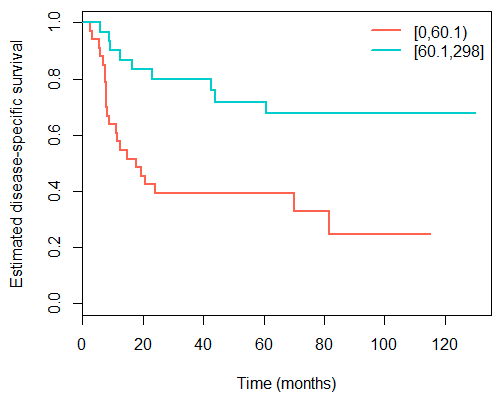


**C)**


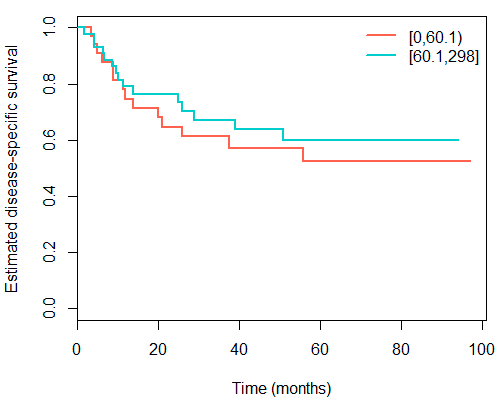


**Figure S4: Kaplan-Meier survival curve of CK20 staining in: A) cystectomy and B) RT 1995-9 and C) RT 2002-5 cohorts split by median.**
